# Patient-controlled admission contracts: a longitudinal study of patient evaluations

**DOI:** 10.1186/s12913-020-06033-4

**Published:** 2021-01-07

**Authors:** Olav Nyttingnes, Jūratė Šaltytė Benth, Torleif Ruud

**Affiliations:** 1 Division of Mental Health Services, Akershus University Hospital, PB 1000, 1478 Lørenskog, Norway; 2 Health Services Research Unit, Akershus University Hospital, Lørenskog, Norway; 3 Institute of Clinical Medicine, University of Oslo, Oslo, Norway

**Keywords:** Mental health care, Inpatient care, Patient autonomy, User participation, Severe mental disorders, Admission procedures

## Abstract

**Background:**

Mental health professionals usually decide patients’ access to inpatient care to ensure care based on need and potential benefit. The purpose of the current study is to investigate how patients evaluate admissions under a contract of Patient-Controlled Admissions (PCA), where the patient could initiate 5 day stays at a community mental health center at their own discretion.

**Methods:**

Patients with a PCA contract in 2011 and 2012 were invited to participate in the study. Staff first recorded clinical baseline values for patients. Towards the end of each PCA stay, staff conducted a structured discharge interview of the admission with the patient. A structured follow-up interview evaluating the PCA arrangement 2 years after inclusion was also performed. We report frequencies from data on PCA requests, PCA admissions and the 2 year evaluation interview, and we used multiple regression models to explore predictors of perceived helpfulness and improvement from the PCA admissions.

**Results:**

The included patients (*n* = 74) made 628 requests for PCAs during the 2 years after inclusion, and 507 PCAs took place. The five-day limit could not be upheld in 7.5% of PCAs. Patients rated PCAs as helping considerably (33.1%), a good deal (30.4%) or somewhat (21.1%), and reported feeling considerably (15.2%), a good deal (26.2%) or somewhat (36.3%) better during the admission. Significant predictors of helpfulness and feeling better were socializing more during the stay and reporting higher motivation to get away from a difficult situation or getting to the ward safety and calmness. A diagnosis of schizophrenia spectrum or bipolar disorder and more services from mental health specialist care also predicted feeling better during the PCA. In the two-year follow-up interview, 90% rated themselves as very or quite satisfied, and more than 90% would recommend PCAs to others.

**Conclusions:**

The PCA arrangement was feasible and was frequently utilized by patients. Patients were satisfied with PCAs and the PCA arrangement. These short stays seemed particularly helpful for patients with a more severe diagnosis. Strong patient satisfaction gives reasons for testing and implementing increased patient influence on the mental health admission procedures in the form of PCAs.

**Supplementary Information:**

The online version contains supplementary material available at 10.1186/s12913-020-06033-4.

## Background

In mental health care, admissions are commonly decided by professionals. This gatekeeping seems necessary to prioritize the patients with the greatest need or high potential benefit. But this approach is not without possible downsides: failing to recognize the true need of the patient, learned helplessness, or power relations with negative effects [1]. These problems give reason to look for other ways to administer inpatient intake, possibly finding ways to facilitate patient influence and empowerment. Access to care when experiencing subjective need is an important priority for many people [2], and influence over admissions could satisfy this need and lead to increased personalization of mental health care.

In 2005, the Jæren Community Mental Health Centre (CMHC) in Southwestern Norway introduced and studied the effect of patient-controlled admission (PCA) contracts. In short, the PCA arrangement invites patients well known in a mental health ward to sign a contract that gives them access to short PCAs by calling the ward and asking for it. PCA stays are typically a maximum of 5 days, followed by a 14 day PCA readmission restriction period, where another PCA is not yet possible [1]. Some variations; omitting the 14 day quarantine or to allow a PCA to last 7 days, were present in some areas in a Danish study [3]. The result of the PCA arrangement in pre-post studies (mirror image studies) is typically that patients on contract increase their number of admissions, but the PCAs are so much shorter that the number of inpatient days drops sharply when compared to a similar period before signing the PCA contract [4, 5]. However, in these designs, regression to the mean cannot be ruled out as the only cause of the changes [6]. A small RCT [5] and a propensity-matched study of PCAs [3] showed a significant reduction in inpatient days in both controls and PCA patients. In these studies, PCA use was lower or the inpatient days reduction was smaller, indicating differences in service or implementation compared to previous Norwegian studies [6]. In any case, none of the published studies have yet reported increased use of inpatient days or other important problems for the patients or wards following PCA introduction, thus indicating non-inferiority for PCAs [6].

Patient preference and satisfaction are important aspects for any health service. Hitherto, patients have expressed strong satisfaction with the freedom, safety, and control provided by PCA contracts, even patients who did not initiate any PCA [7]. Patients signing PCA contracts wanted to get access to early help, avoid admissions at emergency units, and avoid getting very ill or having a long admission. PCAs were initiated after symptoms increased, and in connection with social or practical problems, or to relieve family carers [8]. Such positive results should make PCAs an interesting option, especially in light of the non-inferiority of PCAs regarding the number of inpatient days.

Akershus University Hospital Health Trust piloted a similar PCA arrangement in one CMHC [9]. When later implementing the PCA arrangement in all four CMHCs in the health trust, we arranged a larger pre-post PCA study, and reported large reductions in inpatient days [6]. We also decided to evaluate the PCA arrangement, combining information on each PCA recorded by staff and patients, as well as an overall patient evaluation of the PCA contract in a follow-up interview after 2 years on contract.

The aims of the study were to examine the following research questions: When do patients request a PCA, and what is their motivation? How are the PCAs used? How are the PCA stays evaluated by patients, and how do patients evaluate the PCA arrangement?

## Methods

This is a longitudinal follow-up study of patients’ evaluations of a PCA stays and their evaluation of the patient-controlled admission contract over 2 years. There was no control group. Except for the PCA contracts and PCA stays, the patients received treatment as usual.

### Study context

The study context has been described elsewhere [6]. In brief, Norwegian mental health services are public, with free inpatient services and 79 mental health beds per 100,000 adult inhabitants in Norway in 2014 [10]. The catchment area of Akershus University Hospital has approximately 500,000 inhabitants. There are acute wards, combined high-security and forensic unit and addiction wards at the hospital level. In addition there are four CMHCs with open-door inpatient wards, outpatient services and specialist teams. Two of the CMHCs had used the PCA arrangement before the study started.

### Intervention

Each of the four participating CMHCs reserved two beds for PCAs, about 6% of CMHC beds and 3% of all psychiatric beds for adults in the hospital and CMHCs. PCA contracts were offered to patients who were well known in the ward and with a recent history of admissions to inpatient mental health care. The contract granted the patient access to a stay of a maximum of 5 days based on the patient’s own discretion, with a 14 day period after discharge before a new PCA was allowed. During 2 years, the maximum possible number of 5-days PCAs + 14 day quarantine would be 38. Medications or treatment were not supposed to be changed during the PCA. A call for a PCA could be made between the hours of 09:00 and 20:00, even during weekends. If the dedicated PCA beds were occupied, staff asked the patient to call back later, or they could go to their general practitioner or local casualty clinic for a regular admission.

### Recruitment

Patients signing or having a PCA contract at the four CMHCs were invited by the staff to participate in the study during the years 2011 and 2012. Written informed consent was obtained from those wanting to participate in the study, and the treatment was not influenced by whether or not the patient participated in the study. The study followed up each participating patient for 2 years after their recruitment date. Information was not recorded for patients declining to participate in the study.

### Sample and data collection

**Fig. 1:**
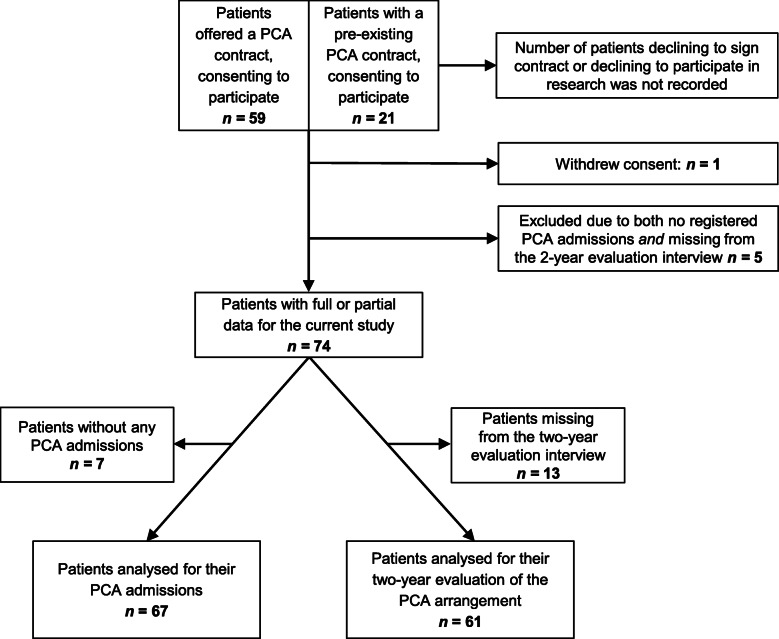
Recruitment, consent, withdrawal, missing data, and analyzed samples

Eighty patients gave written consented and were included in the study. A subset of 59 of these patients signed a PCA contract for the first time at inclusion, and sample characteristics and the change in inpatient days for these 59 patients are described in a previous paper [6]. One of the 80 patients later withdrew consent, and we excluded five patients where there were neither PCAs nor a final evaluation interview. The final sample consists of the 74 remaining patients, and data collected from these are included in the data analyses. Seven patients had no PCA admissions and 13 were missing from the two-year evaluation interview (see Fig. 1). The 74 patients made a total of 628 requests for PCAs, resulting in 507 recorded PCAs. For 59 of these PCA stays, there was no discharge interview recorded, and we report this as missing data in the relevant tables.

### Measurements

At inclusion, staff at the CMHC recorded patient characteristics, including sex, age, living situation, main ICD-10 diagnosis [11], alcohol and substance use in the previous 6 months [12], and Health of the Nation Outcome Scales (HoNOS) for adults. HoNOS is a 12 scale clinician-rated measure of mental health, relations, and functioning, where scores of 3 and 4 indicate need for intervention [13], which we consider the most relevant feature in the sample description. All staff members had been trained in scoring the HoNOS scale. The practical application and response of all staff scoring of all items was discussed and clarified in quarterly project meetings.

Following each request for a PCA, staff filled in a form with information on the PCA request and time of admission and discharge, and finalized the form at discharge, by conducting a structured interview with the patient (see Supplementary file 1 for an English version of the interview form). The interview form covered custom made items on precursors and motivations, social activities during the PCA stay, and evaluation of the stay, based on findings from previous reports [4, 7, 14] and experience from the Ahus PCA pilot study [9]. Seven items were constructed to measure whether patients wanted a PCA to get away from difficulties, symptoms, and have more safety and rest (push), or wanted to get company, activities or structure of the day (pull). The quarterly meetings with staff, indicated that the structured interviews were feasible and meaningful, and that patient responses were not at odds with characteristics of the stays or other patient sentiments available to staff. The measures of primary interest were patient reports of their PCA initiation, their motives for PCAs, and their satisfaction with PCA stays and the PCA arrangement. When a patient had been enrolled in the project for 2 years, staff contacted the patient for a structured evaluation interview, covering the PCA arrangement (see last part of Supplementary file 1 for an English version of this interview form). We based the questions and forms on the experience from the pilot study [9] and the aims of the current study.

### Statistical analysis

We used SAS v9.4 to estimate the regression models, and SPSS v25 for the remaining statistics.

Patient characteristics and requests and admissions under the PCA contract are presented as frequencies and percentages.

First, we analyzed the requests and use of PCAs as single events. This means patients with high use of admissions weigh stronger in these results than patients with fewer admissions. Then we studied the validity of the hypothesized push and pull motivation using a principal component analysis and direct Oblimin rotation. Mean scores of the push and the pull factor items were calculated for each admission.

Predictors for two ordinal satisfaction items; how much the admission did help (helpfulness), and how much better patients got from the admission (improvement), were assessed by bivariate and multiple regression models. The models included random intercepts for patients nested within site. Site effect was assessed by intra-class correlation coefficient (ICC), and if negligible, not adjusted for in the models. We used ordinal regression where the proportional odds assumption was met and a nominal regression model when this assumption was violated. Predictor variables were patient age, sex, main diagnosis, living alone, mean HoNOS score, and the use of municipal and specialist mental health services at baseline. For each admission, we entered the mean push and pull motivation and the degree of contact with ward staff and of social activity during the admission. We also performed a post hoc nominal multilevel regression analysis assessing the change in the patients’ social activity as a function of the patients’ number of admissions.

Finally, we analyzed the two-year evaluations, where each patient’s evaluation is weighed equally, regardless of their use of PCA.

## Results

The patient characteristics are presented in Table 1. A majority of participants were women, and for 48.6% of the patients, the main diagnosis was a psychotic or bipolar disorder (F20–31). The HoNOS scores indicate that the most frequent problems in the sample at inclusion were depressed mood and social relationships with others. One-fourth of the sample needed intervention against hallucinations and delusions, or self-injury, or for improved activities of daily living. Participants used several services; 65 participants (87.8%) used one or more of the listed municipal mental health care services, and 39 (52.7%) used one or more of the outpatient mental health specialist care services.

**Table 1:** Patient characteristics at the time of inclusion (*n* = 74)

	***n***	***%***
*Sex*		
Female	42	56.8
Male	31	41.9
Missing	1	1.4
*Age*		
20–29	8	10.8
30–39	16	21.6
40–49	24	32.4
50–59	21	28.4
60 or above	5	6.8
*Marriage/cohabitating*		
Married or living with a partner	14	18.9
Unmarried, widowed or divorced	60	81.1
*Main diagnoses from the patient record (ICD-10 codes*^*a*^*)*		
Addiction disorder (F10–19)	4	5.4
Schizophrenia spectrum disorder (F20–29)	22	29.7
Bipolar disorder (F30–31)	14	18.9
Depressive disorder (F32–33)	8	10.8
Anxiety and adjustment disorders (F41–43)	11	14.9
Personality disorder (F60–69)	10	13.5
Other disorders	2	2.7
Missing	3	4.1
*Alcohol use*		
Abstinent or non-detrimental use	48	64.9
Abuse or dependency	12	16.2
Missing	14	18.9
*Drug use*		
Abstinent or non-detrimental use	49	66.2
Abuse or dependency	10	13.5
Missing	15	20.3
*HoNOS*^b^ *score 3 or 4 (need for intervention)*		
H01. Overactive, aggressive, disruptive or agitated behavior	4	5.4
H02. Non-accidental self-injury	19	25.7
H03. Problem drinking or drug-taking	13	17.3
H04. Cognitive problems	12	16.2
H05. Physical illness or disability problems	13	17.3
H06. Problems associated with hallucinations and delusions	19	25.7
H07. Problems with depressed mood	37	50.0
H08. Other mental and behavioral problems	36	48.6
H09. Problems with relationships	35	47.3
H10. Problems with activities of daily living	21	28.4
H11. Problems with living conditions	1	1.4
H12. Problems with occupation and activities	8	10.5
*Housing*		
Ordinary housing	60	81.1
Housing with part-time supervision	5	6.8
Housing with full-time supervision	6	8.1
Missing	3	4.1
*Use of municipal care services*		
Municipal day activity center one or more days per week	41	55.4
Face-to-face contact once or more per month	57	77.0
Home services once or more per week	22	29.7
*Use of specialist care services*		
Outpatient consultation once or more per month	29	39.2
Regular contact with psychosis team	16	21.6

^a^ICD-10: International Statistical Classification of Diseases and Related Health Problems, 10th Revision ^b^HoNOS: Health of the Nation Outcome Scale

### Use and evaluation of PCA stays

In the first 2 years after each patient joined the project, staff registered information on 628 requests for PCAs. The timing and results of these requests are presented in Table 2. Almost 25% of PCA requests were made on Mondays, while the number was below 10% on Saturdays and Sundays. The hours with most frequent requests were from 08:00 to 09:59 (30.3% of requests), and 3.3% of requests were made at night (23:00 to 07:59). While 121 requests (19.3%) did not result in admission on the day of the request, staff often asked the patient to call back the next day, and for 67 of the requests without a PCA, a subsequent request resulted in a PCA within a week from the initial request, sometimes after several requests. Another 17 requests were followed by a PCA in the second week following the initial request, leaving 37 requests (5.9% of all requests) without a subsequent PCA within 2 weeks.

**Table 2:** Requests for PCAs (*n* = 628)

	***n***	***%***
*Day of request*		
Monday	155	24.7
Tuesday	86	13.7
Wednesday	81	12.9
Thursday	92	14.6
Friday	93	14.8
Saturday	59	9.4
Sunday	62	9.9
*Time for request*		
Morning (08 through 11)	289	46.0
Afternoon (12 through 16)	208	33.1
Evening (17 through 22)	95	15.1
Night (23 through 07)	21	3.3
Missing	15	2.4
*Result of the request*		
Admission	507	80.7
Rejected because of the 14 days readmission restriction period	5	0.8
Rejected because of no available bed	92	14.6
No admission for other reasons^a^	24	3.8

^a^Most frequent other reasons noted by staff were that the patient canceled the admission, called outside of the contracted time of day for requests, or staffing or capacity problems other than lack of an available bed

Results from patient evaluations at the end of the PCAs are presented in Table 3. Patients described most of the requests that resulted in admissions as uncomplicated. Sixty-three percent of admissions were based on the patients’ own initiative; it was not difficult to ask for the admission in 51.7% of the cases, and they asked for the admission as soon as they needed it in 48.7% of admissions. For a majority of admissions, patients reported that the short PCA stays helped a good deal or considerably, and more admissions were of at least sufficient length (49.1%) than were admissions the patients would have liked to be longer (38.1%). The number of PCA requests per patient ranged from 0 to 50, with a mean (median) of 8.3 (7.0). The number of admissions ranged from 0 to 32, with a mean (median) of 6.7 (4.5).

**Table 3:** Patients’ evaluations of the PCA stay (*n* = 507)

	***n***	%
*Initiative for admission by self or others*		
Realized myself that I wanted an admission	320	63.1
Others suggested an admission for me	115	22.7
I felt pressured by others to admit	9	1.8
Missing	63	12.4
*Was the decision to admit difficult to make?*		
It wasn’t difficult to ask for this admission	262	51.7
It was a little difficult	121	23.9
It was quite difficult or very difficult	63	12.4
Missing	61	12.0
*Do you think you waited too long to ask for an admission?*		
I asked for it when I needed it	247	48.7
I should have asked for it a bit earlier	141	27.8
I should have asked for it much earlier	56	11.0
Missing	63	12.4
*Social activities during the stay*		
Have been with others “a lot” or “partly”	390	76.9
Have had more than one talk with ward personnel	262	51.7
Have had more than one talk with a doctor or psychologist	73	14.4
*Discharge characteristics*		
Patient discharged according to the PCA	432	85.2
Patient discharged, but wanted a longer stay	11	2.2
Patient transferred to regular admission or different ward	38	7.5
Missing	26	5.1
*Did the admission help you?*		
No, it did not help	19	3.7
Yes, it helped somewhat	107	21.1
Yes, it helped a good deal	154	30.4
Yes, it helped very much	168	33.1
Missing	59	11.6
*Have you gotten better during the stay?*		
No, I have not gotten better	54	10.7
Yes, somewhat better	184	36.3
Yes, a good deal better	133	26.2
Yes, very much better	77	15.2
Missing	59	11.6
*Evaluation of the length of the stay*		
I would have liked to stay longer	193	38.1
The length of the stay was alright	238	46.9
I could have managed with a shorter stay	11	2.2
Missing	65	12.8

For 85% of the PCAs, discharge was made according to the PCA contract and within 5 days. For 7.5% of PCAs, the admission resulted in a transfer to another ward or a regular admission at the CMHC. The mean (median) number of nights per PCA where the patients were discharged to the home was 3.5 (4.0).

The principal component analysis of items on the motivation for the admission resulted in two components with eigenvalues above 1, and items loading above 0.6 on each component confirmed the expected push and pull pattern (see Table 4). The two components explained 58.2% of total variance, and the mean scores of 3.5 and 2.7 were in the upper part of a possible score from 1 to 4. Scores on the two factors showed an inter-component correlation of 0.31. Cronbach’s alpha for the push items was .73, and .66 for the pull items.

**Table 4:** Results of principal component analysis among seven items on Patient-Controlled Admissions

Item: How important has it been for you to …	Component loadings (structure matrix)	
	Push	Pull
… be at a place where you could feel safe	**.81**	−.24
… be at a place where you could calm down	**.79**	.10
… get your mental problems reduced	**.73**	.32
… get away from a difficult situation	**.64**	.33
… join in activities	.26	**.81**
… be together with others	.31	**.79**
… get your day more structured	.19	**.72**
Mean score	3.53	2.74

The results of ordinal and nominal regression models are reported in Table 5. The PCA stay helpfulness outcome satisfied the assumption of proportional odds. There was no site effect (ICC = 0), and hence no adjustment included, while the within-patient correlations were notable (ICC = 0.29) and adjusted for. In the multiple model, none of the baseline variables predicted odds for rating the helpfulness item with a higher score. Taking part in more social activities during the stay as compared to each of the three answers reflecting less socializing, or having a higher push or pull motivation for this PCA, was associated with higher odds for scoring PCA helpfulness higher. The multiple model explained 30.2% of the variance in patient-reported helpfulness.

**Table 5:** Multiple ordinal regression model for patient-reported admission helpfulness and nominal regression model for improvement during admission (*n* = 424)

Covariate	Ordinal regression model for the admission helpfulness		Nominal regression model for patient reported improvement during the admission^a^					
			No, don’t feel better		Yes, somewhat better		Yes, a good deal better	
	OR (95% CI)	*p*-value	OR (95% CI)	*p*-value	OR (95% CI)	*p*-value	OR (95% CI)	*p*-value
Age group								
20-29	0.61 (0.15; 2.43)	0.485	0.10 (0.01; 1.47)	0.093	0.72 (0.13; 4.16)	0.714	0.27 (0.05; 1.39)	0.117
30-39	0.72 (0.22; 2.38)	0.591	0.91 (0.14; 5.89)	0.921	0.79 (0.18; 3.49)	0.758	0.31 (0.08; 1.21)	0.091
40-49	0.77 (0.28; 2.12)	0.615	5.53 (1.10; 27.84)	**0.038**	1.57 (0.39; 6.27)	0.520	0.68 (0.19; 2.40)	0.551
50-59 – ref	1		1		1		1	
60+	0.32 (0.07; 1.54)	0.157	5.19 (0.31; 86.77)	0.250	5.30 (0.63; 44.81)	0.125	0.13 (0.01; 1.96)	0.141
Sex, Male	1.04 (0.49; 2.22)	0.913	1.07 (0.35; 3.26)	0.904	1.94 (0.75; 5.04)	0.172	1.65 (0.69; 3.96)	0.260
Living alone, dichotomized – ref “yes”	1.21 (0.44; 3.34)	0.719	0.11 (0.02; 0.71)	**0.021**	0.33 (0.09; 1.15)	0.081	0.34 (0.10; 1.15)	0.083
Diagnosis group								
Other – ref	1		1		1		1	
Schizophrenia	1.75 (0.69; 4.46)	0.243	0.15 (0.03; 0.69)	**0.015**	0.38 (0.11; 1.27)	0.115	0.98 (0.31; 3.08)	0.977
Bipolar disorder	1.30 (0.43; 3.89)	0.643	0.13 (0.02; 0.90)	**0.039**	0.28 (0.07; 1.19)	0.084	0.85 (0.23; 3.10)	0.805
HoNOS mean score	0.79 (0.35; 1.80)	0.575	1.27 (0.38; 4.23)	0.694	0.60 (0.22; 1.64)	0.319	0.72 (0.29; 1.81)	0.484
Sum of municipal services	1.00 (0.66; 1.52)	0.996	1.56 (0.79; 3.07)	0.197	0.85 (0.48; 1.51)	0.581	0.87 (0.50; 1.50)	0.608
Other specialist mental health services, dichotomized – ref 1 or 2	1.75 (0.74; 4.16)	0.204	0.16 (0.04; 0.73)	**0.018**	0.33 (0.11; 0.98)	**0.047**	0.84 (0.30; 2.34)	0.745
Number of patient-controlled admissions	1.01 (0.95; 1.07)	0.695	1.01 (0.94; 1.09)	0.791	0.99 (0.92; 1.06)	0.738	0.97 (0.91; 1.04)	0.409
How much have you been with others during the stay								
I stayed for myself all of time	0.06 (0.02; 0.19)	**<0.001**	31.11 (2.59; 373.49)	**0.007**	9.29 (1.27; 67.94)	**0.029**	0.40 (0.03; 6.00)	0.507
I have been a little together with others	0.22 (0.11; 0.44)	**<0.001**	27.03 (5.69; 128.46)	**<0.001**	4.08 (1.44; 11.56)	**0.009**	1.85 (0.68; 5.08)	0.232
Have been partly together with others	0.42 (0.23; 0.75)	**0.004**	5.26 (1.35; 20.49)	**0.018**	2.99 (1.26; 7.06)	**0.014**	1.84 (0.82; 4.12)	0.143
Have been a lot together with others – ref	1		1		1		1	
Talks with ward personnel, dichotomized – ref ”yes”	0.76 (0.45; 1.34)	0.363	0.34 (0.09; 1.30)	0.116	0.70 (0.29; 1.67)	0.421	0.95 (0.41; 2.23)	0.913
Mean push-motivation for admission	2.46 (1.57; 3.85)	**<0.001**	0.20 (0.07; 0.56)	**0.002**	0.33 (0.14; 0.78)	**0.012**	0.51 (0.21; 1.22)	0.131
Mean pull-motivation for admission	1.58 (1.10; 2.26)	**0.014**	0.38 (0.18; 0.89)	**0.014**	0.59 (0.34; 1.05)	0.073	0.75 (0.43; 1.31)	0.310

*ref* Reference category

^a^The reference category for this outcome variable was “Yes, much better”

For the outcome variable of improvement – whether the patient reported feeling better following the admission – we estimated a nominal regression model (see right columns of Table 5), with random effects for patients nested within sites (ICC = 0.27 and 0.07 at patient and site level, respectively). In the multiple model, push and pull motivation and social activities during the stay significantly predicted odds for reported improvement. The odds for feeling “much better” after the PCA compared to the odds for “not better” or “a little better” were significantly lower if the patients had a lower push motivation or rated their own socialization during the PCA as staying “by myself all the time,” “a little,” or “part of the time” with others compared to “a lot with others.” Lower pull motivation was associated with lower odds for feeling “much better” as compared to “not better.” Among variables collected at baseline, being in their fifties as compared to forties, not living alone, and having a diagnosis of schizophrenia or bipolar disorder as compared to other diagnosis predicted lower odds of rating improvement with the lowest score compared to the highest. Patients regularly receiving other mental health specialist services at baseline had significantly lower odds of rating admission improvement with one of the two lowest scores as compared to the highest.

A post hoc nominal regression model, assessing whether the patients’ socializing during the PCA was associated with the number of admissions, showed a significant non-linear relationship. As compared to “stayed by myself during the stay,” odds for “a little with others,” “part of the time with others,” and “a lot with others” increased with the increasing number of admissions up to about the tenth PCA. After the tenth PCA, odds for more socializing decreased but remained significantly higher than odds for staying “by myself” with increasing number of PCAs (see Supplementary file 2 for a table and figure with parameters and illustration of the results of the model). The four sites differed in how much socializing their patients tended to report during PCAs, with an ICC for site of 0.18.

### Evaluation of the PCA arrangement

**Fig. 2:**
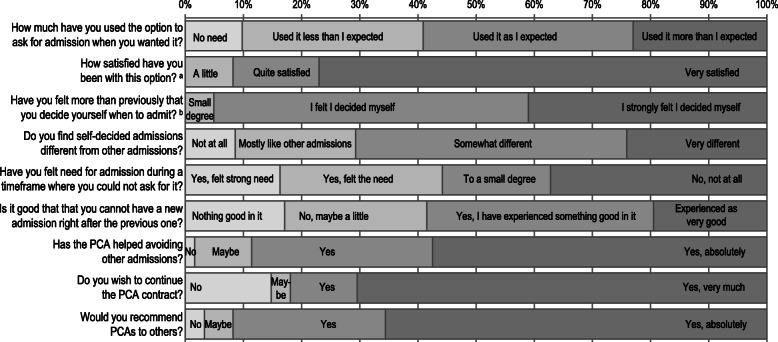
Patient evaluation of the patient-controlled admission arrangement 2 years after enrollment. Sixty-one patients were available for this follow-up, and they left a total of 37 answers missing (6.7%). ^a^ The leftmost alternative; “I have not been satisfied with this offer” was not chosen by any patient. ^b^ The leftmost alternative; “No, not at all” was not chosen by any patient

The results of the patient evaluation interviews after 2 years are shown in Fig. 2. More than 90% of responses indicated satisfaction with PCAs and would recommend the arrangement to others. A strong majority (88%) of the 61 patients available for this evaluation reported that the PCA helped them avoid other admissions and that they felt they were deciding the admission themselves more than previously. For items covering the 14 day readmission restriction period, more than 40% of responses indicated some problems with this limitation, and 44% had felt the need or strong need for admission during such a 14 day period.

## Discussion

The results indicate that the PCA arrangement worked as planned for most of the patients and most of the admissions. Patients initiated many PCAs, and they complied with the upper limit of a five-day stay in a great majority of PCAs. Less than 1 % of requests were rejected because of the 14 day readmission restriction period. Most patients used far fewer PCAs than the maximum possible 38 admissions that the contract allowed during 2 years, although a few patients had from 20 to 32 PCAs during the two-year observation period, and the theoretical maximum would be 38 PCAs of 5 days followed by 14-day readmission restriction during 2 years. In most instances, patients followed the restrictions for timing the requests, with only 3.3% of requests made at night. The higher frequency of requests in the early morning may reflect accumulated need following the night hours. Similarly, the higher number of requests on Mondays may reflect accumulated need after the weekends. We have no data on whether fewer requests on Saturdays and Sundays reflected better mood or mental health during the weekends, a less attractive ward with the reduced weekend staffing, or simply some degree of respect for regular office days and hours. Staff at the CMHCs told us that some patients with part-time child custody used PCAs after weekends where they had focused on and tended to their offspring. The wards were able to give a PCA on the same day for 80% of the requests, and 6 % of the requests did *not* result in a PCA within 2 weeks. These findings confirm previous studies from Norway and Denmark, where the PCA arrangement is described as feasible and acceptable for a large part of the included patients [3, 9, 14, 15].

A majority of requests were initiated by the patients themselves, and decided when they felt the need. Both push and pull motivation factors – wanting the PCA to get away from a difficult situation *and* to get company, activities, and structuring the day – were important for patients, with a clear factor structure and a particularly high mean score for the push factor. The push and pull motivation factors correlated with .31, meaning that while some PCAs were primarily motivated by one of the factors, in other cases, the patient had both push and pull motivation for the PCA. These findings are in line with the Danish PCA evaluation, where around half of the admissions were solely based on the patient’s decision, and a variety of motives for admission included increased social and practical problems and having contact with staff and getting structure [8].

In spite of the strongly restricted length, admissions were commonly evaluated as helpful, with only 3.7% of PCAs evaluated as not helpful, and patients reported no improvement after 10.7% of PCAs. Also, the two-year evaluation of the PCA arrangement was strongly positive, with over 90% of patients being satisfied with the option and recommending it to others. While some of the patients wanted some PCAs to be longer, the five-day limit for a PCA appeared sufficient for a great many PCAs in our study, also in line with previous findings [3, 15]. We have no control group to compare with, but at face value, the approval of PCA appears strong. PCAs were required to be short and the content does not deviate much from regular admissions. This indicates that the shifting of gatekeeping powers to the patient and away from professionals and bureaucratic procedures is important. This fits well with the documented importance to be in control of aversive events, that clinical populations have lower level of control than non-patients [16], and that patients report safety as the core of the PCA benefit [17]. The favorable evaluation of PCAs in our study questions whether the possible gains from professionals symptom evaluations in the gatekeeping process should be considered more important than the possible gains from giving mental health patients more safety through controlling their own admissions.

A great majority of PCAs were reported as helpful, and one-third of admissions were given the highest score (helped considerably). According to the ICC, the level of helpfulness did not vary between sites, indicating that all sites provided a similar level of helpfulness. There were notable within-patient correlations, indicating that each patient tended to report a consistent level of helpfulness, which could vary from patient to patient. Patients who socialized more during the PCA and reported higher push or pull motivation found the admission significantly more helpful, while none of the patient characteristics collected at baseline was significant in the multiple model. The sites differed somewhat in how much their patients socialized during PCAs, and patients tended to socialize more during later admissions. This might result from increased familiarity with the ward and the PCA arrangement, or after learning how to best achieve satisfying PCAs. The group of patients that used more than ten PCAs during the observation period seemed to have more problems with socializing during their stays, and this contributed to the curvilinear effect of repeated PCAs on the degree of patient socializing. It is reasonable to think that more socializing stimulates improvement, but stable differences in patients’ social style may also confound the observed relation. A recent qualitative study of patients with a PCA-contract found that they felt relating with staff were helpful, and if they felt ignored or pushed aside, they tended to withdraw from contact with staff [17]. This indicates that emphasizing and strengthening patient socializing might indeed improve outcome.

Patients also rated themselves as improved at the discharge from a great majority of PCAs, and socializing more during the stay and having a higher push or pull motivation predicted lower odds for a lower score, in line with the results for helpfulness. Nevertheless, for improvement, indicators of severity, such as a more severe diagnosis (schizophrenia or bipolar disorder) and receiving more specialist mental health services at baseline, also predicted lower odds for a low score. This indicates a better outcome in the eyes of patients with such indicators at baseline, compared to those with less severe scores. These baseline scores may have changed during the two-year period, but findings nevertheless indicate that high initial severity should not rule out engaging the patient in a PCA contract. While the level of improvement varied somewhat between sites, the effect was small. The high satisfaction with PCAs in this study is in line with high satisfaction reported in other qualitative and quantitative studies [7–9, 17, 18]. It is noteworthy that patients with more severe diagnosis and more specialist care at baseline found themselves so firmly helped by a self-initiated 5 day stay. While the possibility of timing the admission to self-perceived need should be similar for all PCA patients, the increased agency and safety following control over the admission may be a larger change from regular gatekeeping of admissions for those with a more severe baseline situation.

Patients reported strong satisfaction with the PCA arrangement in the two-year evaluation. The results corresponded to the intentions regarding the arrangement; more than 90% felt more than before that they decided when to admit, and 88% responded that the PCA had helped them avoid other admissions. On the other side, they expressed mixed evaluations of the 14 days readmission restriction, where less than 40% had not felt need for admission when they could not ask for it, and only 20% rated this part of the arrangement as very good. The least popular aspect of the arrangement thus seemed to be where the gatekeeping powers remained with the professionals. The Danish evaluation also reported mixed evaluations of the readmission restriction, where 40% of patients worried about the day limit, and 21% found the stay too short [8].

### Strengths and limitations

The study collected data from four CMHCs over a two-year period, indicating that the positive patient evaluation is not based on a few dedicated clinicians or an initial post-contract positivity boost. The measurements were mostly custom-made questions in structured interviews conducted by staff. Questions were closely adapted to the patient’s situation, but the scores are not validated. When patients were interviewed by clinical staff, it raises the issue whether demand characteristics have contributed to the very favorable rating of PCAs. The combination of collecting data on patient evaluation of single PCAs and the PCA arrangement after 2 years strengthens the conclusions of strong patient satisfaction with PCAs. Findings in this study are from the subjective perspective of the patients, and we did not collect family carers or provider evaluations. We have previously reported changes in inpatient day use for a subsample of this study [6]. We had no control group rating regular admissions, so we could not make comparisons to treatment as usual. Also, no measure of symptom or functioning was collected during the PCAs, precluding a closer examination of whether the clinical situation during the PCA influenced patient reported satisfaction or improvement. The study is from one hospital area in a small country, which limits the generalizability of the findings. Some care setting variables, such as beds per capita, local admission procedures, and the level of alliance and cooperation in the area, may be important for the results and limit generalizability to areas with different characteristics. The recruitment process demanded an overall clinical evaluation of suitability for PCAs, which may have biased our sample towards alliance and satisfaction, compared to the full group of CMHC patients or mental health inpatients.

## Conclusion

PCA use showed that the PCA framework is feasible, and patients reported strong satisfaction with individual PCAs as well as the PCA arrangement. Requests were mostly made on weekdays, within office hours. Patients requested PCAs both as a push from problems in their current living situation, and as a pull towards the company and structure of the ward. They rated PCAs as better than their previous admission experience and felt helped and improved, even after the short stays of a maximum of 5 days. Patients with more severe problems reported more improvement. In light of the indications of reduced inpatient day use in pre-post-studies and the absence of reports of negative effects, the strong patient satisfaction constitutes a reason for implementing and testing PCAs for increased patient influence on the admission decision.

## Supplementary Information

**Additional file 1 **Structured interview forms for the discharge interview and the two year evaluation interview for patients with patient controlled admissions.

**Additional file 2 Supplement table.** Results from a nominal regression model for the patients’ socializing during the patient controlled admission, adjusted for within patient correlations and site effect (ICC=0.18). **Supplement figure.** Illustration of odds for the different levels of the patients’ socializing as a function of the patient’s admission number.

## Data Availability

The dataset analyzed in the current study is available from the corresponding author on reasonable request.

## Abbreviations

CMHC: Community mental health center
CTO: Community treatment order
GP: General practitioner
HoNOS: Health of the Nation Outcome Scales
ICD-10: International Statistical Classification of Diseases and Related Health Problems, 10th Revision
PCA: Patient-controlled admission
