# Supplementary material for: Patient-controlled admission contracts: a longitudinal study of patient evaluations
Source: BMC Health Serv Res. 2021 Jan 7;21:36. doi: 10.1186/s12913-020-06033-4 (PMC7791868; doi:10.1186/s12913-020-06033-4)
Supplement: Supplementary file 1 — Additional file 1 Structured interview forms for the discharge interview and the two year evaluation interview for patients with patient controlled admissions. [file 12913_2020_6033_MOESM1_ESM.docx]

# Structured discharge interview with patients in Patient Controlled Admissions

*Instruction to staff: Tell the patient that his/her experience can help improve the care offers, and we would like to ask some questions regarding the current admission. Read the questions and alternatives, and mark the answer given by the patient (do not read the number, only the text).*

1. **Did others encourage you to ask for an admission?**

| □ | No, I realized myself that I wanted an admission |
| --- | --- |
| □ | Yes, others suggested an admission for me |
| □ | Yes, I felt pressured by others to come here |

**2. Was the decision to admit difficult to make?**

| □ | No, It wasn’t difficult to ask for this admission |
| --- | --- |
| □ | Yes, it was a little difficult |
| □ | Yes, it was quite difficult |
| □ | Yes, it was very difficult |

**3. Do you think you waited too long to ask for an admission?**

| □ | No, I asked for it when I needed it |
| --- | --- |
| □ | Yes, I should have asked for it a bit earlier |
| □ | I should have asked for it much earlier |

**4. Do you think the admission has been of suitable length?**

| □ | No, I would have liked to stay longer |
| --- | --- |
| □ | Yes, the length of the stay was alright |
| □ | Yes, but I could also have managed with  a shorter stay |

**5. How much have you been with others during the stay?**

| □ | I stayed for myself all of the time |
| --- | --- |
| □ | I have been a little together with others |
| □ | I have partly been together with others |
| □ | I have been a lot together with others |

**6. Have you participated in activities with others?**

| □ | No |
| --- | --- |
| □ | Yes, once |
| □ | Yes, a few times |
| □ | Yes, many times |

**7. Have you had talks with the ward personnel?**

| □ | No |
| --- | --- |
| □ | Yes, once |
| □ | Yes, a few times |
| □ | Yes, many times |

**8. Have you had talks with the doctor or psychologist?**

| □ | No |
| --- | --- |
| □ | Yes, once |
| □ | Yes, a few times |
| □ | Yes, many times |

**9. Did the admission help you?**

| □ | No, it did not help |
| --- | --- |
| □ | Yes, it helped somewhat |
| □ | Yes, it helped a good deal |
| □ | Yes, it helped very much |

**10. Have you gotten better during the stay?**

| □ | No, I have not gotten better |
| --- | --- |
| □ | Yes, somewhat better |
| □ | Yes, a good deal better |
| □ | Yes, very much better |

(Turn to the next page)

Say: *“Finally we have a few questions about what has been important for you regarding this stay”*

**How important has it been for you to …**

**11. – get away from a difficult situation**

| □ | Not important or no need for this |
| --- | --- |
| □ | A little important |
| □ | Quite important |
| □ | Very important |

**12. – be at a place where you could feel safe**

| □ | Not important or no need for this |
| --- | --- |
| □ | A little important |
| □ | Quite important |
| □ | Very important |

**13. – be at a place where you could calm down**

| □ | Not important or no need for this |
| --- | --- |
| □ | A little important |
| □ | Quite important |
| □ | Very important |

**14. – get your mental problems reduced**

| □ | Not important or no need for this |
| --- | --- |
| □ | A little important |
| □ | Quite important |
| □ | Very important |

**15. – be together with others**

| □ | Not important or no need for this |
| --- | --- |
| □ | A little important |
| □ | Quite important |
| □ | Very important |

**16. – join in activities**

| □ | Not important or no need for this |
| --- | --- |
| □ | A little important |
| □ | Quite important |
| □ | Very important |

**17. - get your day more structured**

| □ | Not important or no need for this |
| --- | --- |
| □ | A little important |
| □ | Quite important |
| □ | Very important |

**18. Are there other things that have been important to you?**

______________________________________

______________________________________

______________________________________

______________________________________

______________________________________

# The patient’s experience with Patient Controlled Admissions

**1. The form is filled in the following situation:**

| □ | Regular 2 year evaluation |
| --- | --- |
| □ | The patients move to another district and the contract is discontinued before 2 years |
| □ | The contract is discontinued before 2 years because of other reasons |
| □ | The patients has withdrawn from the project |

*We ask you to answer a few questions about how you have experienced the possibility of deciding yourself when you wanted an admission.*

*Your experiences will be used to evaluate how such an offer can be continued or improved.*

Tick the box to the left of the answer chosen by the patient.

1. **How much have you used the option to ask for admission when you wanted it?**

| □ | I have not needed this option |
| --- | --- |
| □ | I have used it less than I expected |
| □ | I have used it as I expected to |
| □ | I have used it more than I expected |

**3. How satisfied have you been with this offer?**

| □ | I have not been satisfied with this offer |
| --- | --- |
| □ | I have been a little satisfied with this offer |
| □ | I have been quite satisfied with this offer |
| □ | I have been very satisfied with this offer |

**4. Have you felt more than previously that you decide yourself when you want an admission?**

| □ | No, not at all |
| --- | --- |
| □ | No, I have felt deciding myself it to a small degree |
| □ | Yes, I felt I decided myself |
| □ | Yes, I strongly felt I decided myself |

**5. Do you find self-decided admissions
different from other admissions?**

| □ | No, not at all |
| --- | --- |
| □ | No, it has mostly been like other admissions |
| □ | Yes, it has been somewhat different |
| □ | Yes, it has been very different |

**6. Have you felt need for admission during a timeframe where you could not ask for it?**

| □ | No, not at all |
| --- | --- |
| □ | No, I have felt the need to a small degree |
| □ | Yes, I have felt the need for it |
| □ | Yes, I have felt a strong need for it |

**7. Is it good that that you cannot have a new admission right after the previous one?**

| □ | No, I haven’t experienced any good in it |
| --- | --- |
| □ | No, but maybe it is a little good in it, too |
| □ | Yes, I have experienced something good in it |
| □ | Yes, I have experienced it as very good |

**8. Has the PCA helped avoiding
other admissions?**

| □ | No |
| --- | --- |
| □ | Maybe |
| □ | Yes |
| □ | Yes, absolutely |

**9. Do you wish to continue the PCA contract?**

| □ | No |
| --- | --- |
| □ | Maybe |
| □ | Yes |
| □ | Yes, very much |

**10. Would you recommend patient controlled admissions to others?**

| □ | No |
| --- | --- |
| □ | Maybe |
| □ | Yes |
| □ | Yes, absolutely |

**11. Are there anything that ought to be different in the contract of patient controlled admissions?**

| □ | Yes |
| --- | --- |
| □ | No |

If answering yes, please tell what should have been different:

___________________________________________________________________________________________________________________________________________________________________________________________________

**12. Are there anything that ought to be different in the patient controlled *admissions* you have had?**

| □ | Yes |
| --- | --- |
| □ | No |

If answering yes, please tell what should have been different:

___________________________________________________________________________________________________________________________________________________________________________________________________

*Thank you sharing your experiences with us!
It can help us improve the care offers.*
