# Supplementary material for: Patient-controlled admission contracts: a longitudinal study of patient evaluations
Source: BMC Health Serv Res. 2021 Jan 7;21:36. doi: 10.1186/s12913-020-06033-4 (PMC7791868; doi:10.1186/s12913-020-06033-4)
Supplement: Supplementary file 2 — Additional file 2 Supplement table. Results from a nominal regression model for the patients’ socializing during the patient controlled admission, adjusted for within patient correlations and site effect (ICC=0.18). Supplement figure. Illustration of odds for the different levels of the patients’ socializing as a function of the patient’s admission number. [file 12913_2020_6033_MOESM2_ESM.docx]

# Supplementary file 2

**Supplement table:** Results from a nominal regression model for the patients’ socializing during the patient controlled admission, adjusted for within patient correlations and site effect (ICC=0.18)

| Covariate | Regression coefficient (SE) | p-value | |
| --- | --- | --- | --- |
| Intercept  Stayed for myself all time during the stay  Have been a little together with others  Have been partly together with others  Have been a lot together with others  Time  Stayed for myself all time during the stay  Have been a little together with others  Have been partly together with others  Have been a lot together with others  Time x Time  Stayed for myself all time during the stay  Have been a little together with others  Have been partly together with others  Have been a lot together with others | 0  0.93 (0.54)  0.75 (0.56)  0.37 (0.61)  0  0.36 (0.18)  0.47 (0.18)  0.43 (0.19)  0  -0.02 (0.009)  -0.02 (0.009)  -0.02 (0.01) | 0.087  0.184  0.549  **0.045**  **0.010**  **0.022**  **0.026**  **0.010**  **0.019** |  |

**
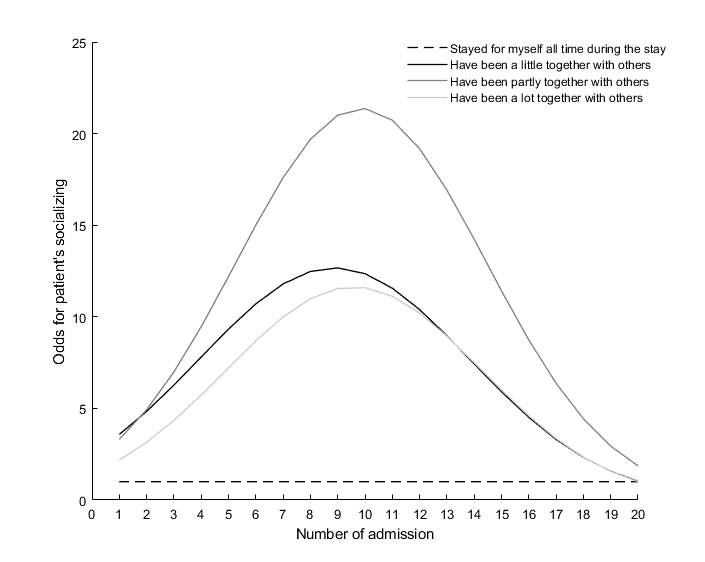
Supplement figure:** Illustration of odds for the different levels of the patients’ socializing as a function of the patient’s admission number.
